# Reduced cortical expression of a newly identified splicing variant of the *DLG1* gene in patients with early-onset schizophrenia

**DOI:** 10.1038/tp.2015.154

**Published:** 2015-10-06

**Authors:** A Uezato, N Yamamoto, Y Iwayama, S Hiraoka, E Hiraaki, A Umino, E Haramo, M Umino, T Yoshikawa, T Nishikawa

**Affiliations:** 1 Department of Psychiatry and Behavioral Sciences, Graduate School of Medical and Dental Sciences, Tokyo Medical and Dental University, Tokyo, Japan; 2 Laboratory for Molecular Psychiatry, RIKEN Brain Science Institute, Wako, Japan

## Abstract

The human discs, large homolog 1 gene (*DLG1*) is mapped to the schizophrenia-susceptibility locus 3q29, and it encodes a scaffold protein that interacts with the N-methyl-D-aspartate receptor presumably dysregulated in schizophrenia. In the current study, we have newly identified a splicing variant of *DLG1*, which is transcribed from an unreported 95-base-pair exon (exon 3b) and is labeled 3b(+). We investigated the mRNA expression of 3b(+) in the post-mortem dorsolateral prefrontal cortices of patients with psychiatric disorders, obtained from The Stanley Medical Research Institute, and examined the potential association of the expression with the genotype of the single-nucleotide polymorphism (SNP) rs3915512 located within exon 3b. A real-time quantitative reverse transcriptase-polymerase chain reaction revealed that the mRNA levels of 3b(+) were significantly reduced in patients with early-onset schizophrenia (onset at <18 years old, *P*=0.0003) but not in those with non-early-onset schizophrenia, early-onset or non-early-onset bipolar disorder or in the controls. Furthermore, the genotype at the rs3915512 SNP was closely associated with the levels of 3b(+) mRNA expression. It is inferred that the T allele fails to meet the exonic splicing enhancer consensus, thus resulting in skipping of exon 3b, leading to the expression of 3b(−) (the previously known *DLG1* variant) but not 3b(+). Because all the subjects with early-onset schizophrenia in the current study possess the T/T genotype, the reduced level of the *DLG1* 3b(+) transcript may be involved in the susceptibility and/or pathophysiology of early-onset schizophrenia.

## Introduction

Schizophrenia is a serious psychiatric disorder with a high prevalence of nearly 1% and a wide variety of mental dysfunctions that are only partially ameliorated by current antipsychotic drugs. It has been widely accepted in recent decades that N-methyl-D-aspartate glutamate receptor (NMDAR) hypofunction may be involved in the pathophysiology of schizophrenia because NMDAR antagonists and anti-NMDAR antibodies cause psychotomimetic effects,^1, 2, 3, 4^ which might provide a valuable clue to understand the molecular and cellular basis of this disorder for development of more effective therapeutic methods. Moreover, it is notable that, similarly to the onset of schizophrenia, schizophrenia-like symptoms elicited by antagonists and antibodies for NMDAR usually occur after, but not before, adolescence in humans and a critical period of postnatal development in experimental animals.^4, 5, 6, 7, 8, 9, 10, 11^ These observations suggest that the psychotic symptoms of schizophrenia and those elicited by NMDAR blockade require the functional maturation of a specific brain system during a critical stage of development. Such a human system and its animal homolog should be equipped with molecules that respond differentially to NMDAR antagonists before and after the critical period. Therefore, we can hypothesize that these molecules could be associated with the onset of schizophrenia.

In our previous studies based on this hypothesis, to screen for schizophrenia-related genes we chose candidate genes that were upregulated in brain tissues by phencyclidine administration after the above critical period, ~3 weeks after birth, but failed to have any effect before this period. Among such genes, we have identified *Dlg1* (discs, large homolog 1 of *Drosophila*)/*SAP97* (synapse-associated protein 97 (*DLG1*)), which encodes synaptic scaffold proteins interacting with ionotropic glutamate receptors including NMDAR.^12, 13^ Indeed, we found a genetic association of schizophrenia with human *DLG1* from our two separate cohort studies using single-nucleotide polymorphisms (SNPs).^14, 15^ In agreement with our results, expression changes in the DLG1 proteins were observed in brains of patients with schizophrenia post-mortem.^16, 17^ Moreover, genome-wide analyses of the copy-number variation found a significant excess of deletions in schizophrenia at the chromosomal position 3q29, which includes the *DLG1* gene.^18, 19^

In the present study, to extend our insight into the pathophysiological role of the *DLG1* gene in schizophrenia, we first explored the complementary DNA (cDNA) library of the post-mortem human cerebral cortex in an attempt to identify unreported *DLG1* splicing variants. Then, we investigated the gene expression of these variants in the post-mortem brain tissues of patients with schizophrenia and bipolar disorder. Furthermore, we examined potential associations between gene expression and specific genotypes. In the data analyses, we especially explored the possible relationship between the *DLG1* gene expression and either the age of onset or the genotype.

## Materials and methods

### Ethical approval

This study was approved by the Ethics Committees of Tokyo Medical and Dental University and RIKEN and was conducted in strict accordance with the guidance of these institutes and the principles expressed in the Declaration of Helsinki.

### Re-examination of the complex *DLG1* transcript variants in the cDNA library of the post-mortem human cerebral cortex

A reverse transcriptase-polymerase chain reaction (RT-PCR) was performed using cDNA derived from pooled post-mortem brain RNAs (TakaraBio/Clontech, Kusatsu, Japan, and SuperScript VILO cDNA Synthesis Kit, Invitrogen, Carlsbad, CA, USA, for reverse transcription). The PCR conditions were as follows: 94 °C for 1 min; one cycle of 94 °C for 2 min, 60 °C for 3 min and 72 °C for 5 min; two cycles of 94 °C for 45 s, 60 °C for 2 min and 72 °C for 3 min; 32 cycles of 94 °C for 30 s, 60 °C for 45 s, 72 °C for 1 min; and 72 °C for 5 min. The primers used were forward primer: 5′-CGGAAGCAAGATACCCAGAG-3′, which corresponded to nucleotide positions 200–219 of the human *DLG1* variant 1 cDNA (NM_001098424) and reverse primer: 5′-AATTGGTTCAGACGGCTTTG-3′, which corresponded to nucleotide positions 399–418 of the above variant 1 cDNA). The PCR product was separated using gel electrophoresis on a 3% agarose gel in 1xTAE; the gel was then dyed for 20 min with 0.5 μg ml^−1^ ethidium bromide and visualized using a ultraviolet transilluminator. Following extraction of the novel PCR product from the agarose gel and TA cloning (pGEM-T Easy Vector, Promega, Madison, WI, USA), the sequence of the amplified cDNA was determined with an autosequencer (ABI PRISM 3100 Genetic Analyzer, Applied Biosystems, Foster City, CA, USA). With the speculation that the novel PCR product reflects a previously unreported splicing variant of *DLG1*, we designed a second primer set to obtain a PCR product that covers the full open reading frame of the novel variant (forward primer: 5′-GGTCACGCCTCTCTTCAGAC-3′, which corresponded to nucleotide positions 72–91 of the *DLG1* variant 1 cDNA; novel variant sequence-specific reverse primer: 5′-TTGAATATCTGACAGGACACACTG-3′, part of which corresponded to nucleotides 342–49 of variant 1, although the other part (3'-side of the primer) was novel (variant-specific).

### Subjects for post-mortem study

The RNA samples extracted from the post-mortem dorsolateral prefrontal cortices (DLPFC; Brodmann area 46) were obtained from The Stanley Medical Research Institute.^20^ They included total RNA samples from 34 schizophrenia patients, 33 bipolar disorder patients and 34 controls without neuropsychiatric disorders. Patients were stratified by onset age before they were 18 years old (early onset) and equal to or older than 18 years old (non-early onset);^21^ thus, the patient groups were early-onset schizophrenia (EOS), non-early-onset schizophrenia (non-EOS), early-onset bipolar disorder (EOBP) and non-early-onset bipolar disorder (non-EOBP; Supplementary Table S1). This stratified analysis according to the onset age potentially would be valuable to reduce the heterogeneity that is considered to be included in schizophrenia because (i) the onset ages of diseases with different causes sometimes vary even if these diseases have similar symptoms, (ii) the developmentally regulated nature of the phencyclidine-induced changes in the expression of the *Dlg1* mRNA variants suggests the possible involvement of the *DLG1* gene in molecular mechanisms underlying the adolescence-related onset of schizophrenia^22^ and (iii) schizophrenia with an onset age under 18 is often classified as EOS for biological and clinical studies.^21^ Because the RNA samples were coded, the diagnoses of the subjects were masked while the assays were being performed.

The demographic details of the subjects and the preliminary real-time quantitative PCR (qRT-PCR) assay of housekeeping genes are shown in Supplementary Table S1. Only a single subject, in the EOBP group, was African-American and all the others were Caucasians; therefore, the effects of ethnicity were not examined in the present study. The presently used post-mortem specimens were judged to preserve the acceptable quality for the qRT-PCR assay of mRNAs because (i) their pH values are within the ranges that have been recognized as being reliable (for example, references^23, 24, 25, 26^), although there is variation within this range, as indicated by the analysis of variance analysis, which showed significant differences in the pH values between the control (CT) and non-EOS groups, and (ii) similar expression levels of the housekeeping genes, *GAPDH, ACTB, PGK1*, 18S and 28S rRNA, observed across all the diagnostic groups, suggest that the samples maintain the homogeneity of their RNA quality of the current sample set.

### Quantitative reverse transcriptase-polymerase chain reaction

The mRNA levels were determined with qRT-PCR using TaqMan Universal PCR Master Mix, transcript-specific minor groove-binding probes (TaqMan Gene Expression Assays, Applied Biosystems) and an ABI 7900 sequence detection system (Applied Biosystems), according to the manufacturer's instructions. The details of the protocols have been described elsewhere.^27, 28^ The TaqMan primer/probes for glyceraldehyde-3-phosphate dehydrogenase (GAPDH; NM_002046), which served as an endogenous reference, were purchased from Applied Biosystems (Assay-on-Demand gene expression products Hs03929097_g1). The primers/probes for the *DLG1* variants were designed to specifically amplify sequences corresponding to the newly identified variant (forward primer: 5′-AGAACGGGTTATTAACATATTTCAGAGCAA-3′, reverse primer: 5′-GAAAGTAGTCTTCAAATCACACCAACAAT-3′, FAM probe: 5′-CAGGCTTTAATAGTAACTTCC-3′) and the already known variant (forward primer: 5′-CAGAAGTTCCATAGAACGGGTTATTAACA-3′, reverse primer: 5′-ATTTTGGATTATCCAGTAAGGTCACTTCAT-3′, FAM probe: 5′-CTTTCAGGCTTTAATAGATATTC-3′). The PCR assay was performed simultaneously for the test and standard samples, and with no template controls in the same plate. A standard curve plotting the cycle of the threshold values versus the input quantity (log scale) was constructed for both the *GAPDH* gene and the target molecules for each PCR assay. All the qRT-PCR data were captured with SDS v2.4 (Applied Biosystems). The ratio of the relative concentration of the target molecule to the *GAPDH* gene (target molecule/*GAPDH*) was calculated to represent the mRNA expression. In the present study, we utilized only the *GAPDH* gene as an endogenous reference because we had already demonstrated by using the same samples that the level of *GAPDH* is linearly correlated with those of other housekeeping genes, *ACTB* and *PGK1* (Supplementary Figure S1).

### Genotyping

The genomic DNA was provided by the Stanley Medical Research Institute. The quality and quantity of the DNA were estimated using a ultraviolet spectrophotometer. Genotypes were determined using Sanger sequencing. In detail, the target region was amplified using the following primers: forward: 5′-CTGGCTCTTTTGTCCAGGTACC-3′, reverse: 5′- ATGTGAATTTCTGGAGCTGGGT-3′. The PCR products were sequenced using the BigDye Terminator v3.1 cycle Sequencing Kit (Applied Biosystems) and an ABI 3730 Genetic Analyzer (Applied Biosystems), using the standard protocols. The variant was detected using the Sequencher software (Gene Codes, Ann Arbor, MI, USA). For the heterozygous variant call in Sequencher, the height of the secondary peak was set at 35% of the primary peak, and the variant was also confirmed by bidirectional sequencing of the sample.

### Statistical analysis

Statistical calculations were performed with IBM SPSS v.23 for Windows (IBM, Armonk, NY, USA). A one-way analysis of variance was used to examine the variability in the distribution of the demographic variables across groups, followed by a *post hoc* analysis with the Tukey Honestly Significant Differences test. Correlations between the mRNA expression and other variables (age, post-mortem interval, brain pH or lifetime dose of antipsychotics) were evaluated by calculating the Spearman rank correlation coefficient because of the heteroscedasticity of the mRNA data. The effect of gender on expression levels of various mRNAs was evaluated using Welch's *t*-test. The *DLG1* variant expression for each diagnostic group was compared with that of the controls, and a statistical analysis was performed using a two-sided Welch's *t*-test with the Bonferroni correction.^29^ We used Welch's *t*-test for these two comparisons because they include heteroscedastic data.

## Results

### Isolation of a newly identified splicing variant of human *DLG1* mRNA

**Figure 1:**
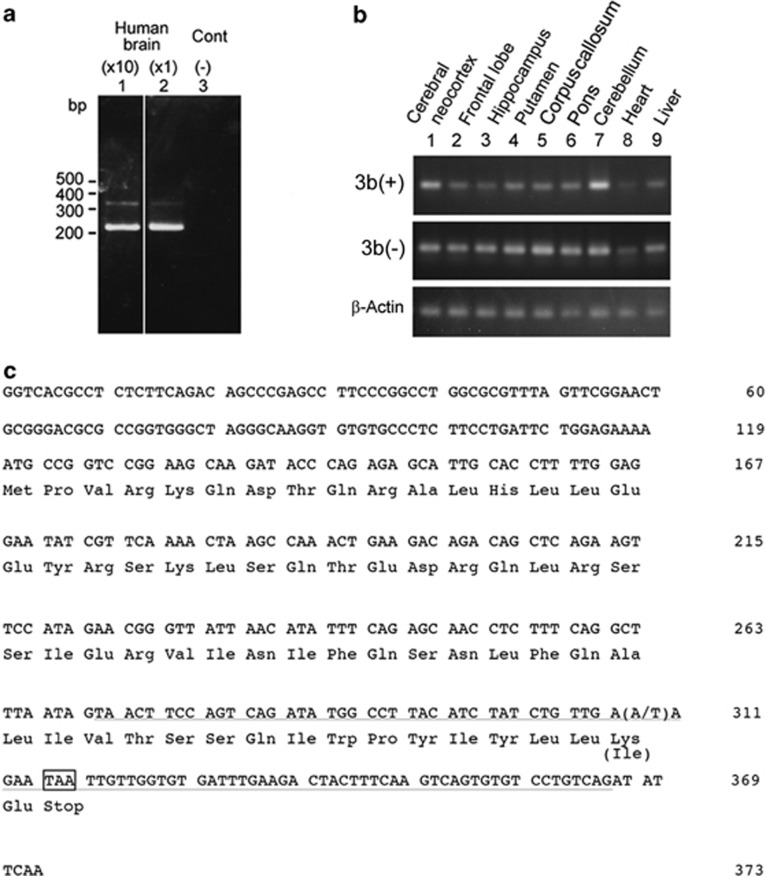
A newly isolated human 3b(+) variant of *DLG1* mRNA. (**a**) Detection of a novel splicing variant with a size of 314 bp, which contains 95 bp of exon 3b, above the major band with a size of 219 bp by reverse transcriptase-polymerase chain reaction (RT-PCR) from the human brain. The former PCR product was confirmed to be detected in different concentrations of complementary DNA (cDNA) templates: the cDNA used in the second lane was 10 times more dilute than that in the first lane. Control (−) indicates PCR without template DNA. Each number (base pairs) on the left indicates the migration position of the 100-bp DNA ladder. (**b**) Distribution of 3b(+) and 3b(−) transcripts in various human brain regions and peripheral organs. The total RNAs from each brain region and peripheral tissue were obtained from TakaraBio/Clontech. (**c**) Nucleotide sequence of the underlined exon 3b transcript and the deduced amino-acid residues of the predicted open reading frame in amino-acid symbols below the respective codons.

**Figure 2:**
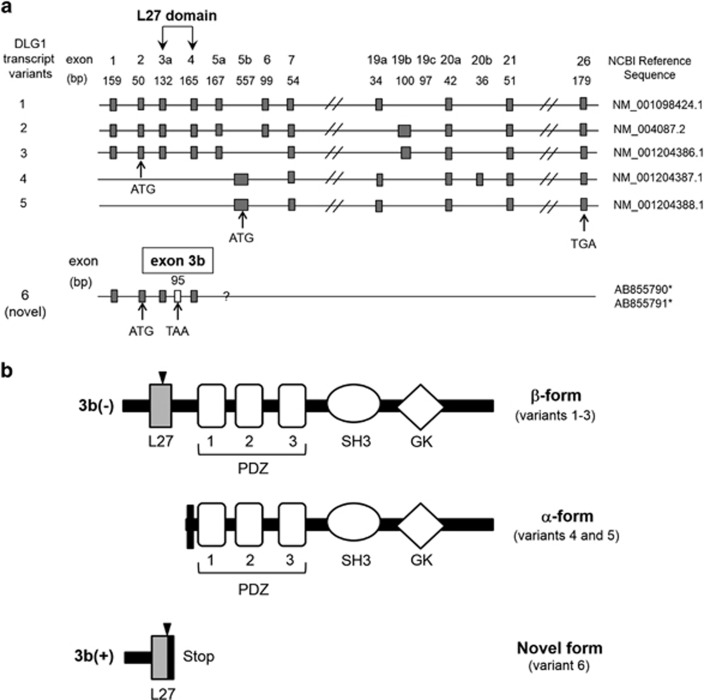
Structures of human *DLG1* transcripts and proteins. (**a**) Schematic representation of the structure of human *DLG1* transcript variants. The insertion of exon 3b between exons 3a (relabeled from original exon 3) and 4 might result in the premature termination of translation because of the in-frame stop codon in exon 3b (see **b**). *The new transcript variants with A and T alleles for rs3915512 were numbered AB855790 and AB855791, respectively. (**b**) Schematic representation of the structure of the putative proteins translated from the *DLG1* transcript variants. The β- and α-form proteins are formed from the transcript variant 1–3 group and variant 4–5 group, respectively. From the transcript variant 6 including the 3b(+) message, a 65-amino-acid-long protein containing 15 unique C-terminal amino acids is formed. The arrowheads indicate the sites of the splicing variants.

We isolated a transcript with an unreported insertion sequence consisting of 95 base pairs (Figures 1a and c, and Figure 2a). The genomic DNA sequencing analysis revealed that the insertion is transcribed from a newly identified exon located between exons 3 and 4 of the *DLG1* gene, which we have designated exon 3b. The transcripts with and without the novel insertion were labeled 3b(+) and 3b(−) mRNA, respectively. Substantial amounts of the 3b(+) mRNAs are widely detected in various human brain and peripheral tissues (Figure 1b).

The deduced amino-acid sequence of the putative protein translated from the 3b(+) *DLG1* transcript variant indicated that the insertion of exon 3b between exons 3a (relabeled from the original exon 3) and 4 might lead to a premature termination of translation due to the in-frame stop codon in exon 3b (Figure 2a). The presumed transcript including the 3b(+) message would form a 65-amino-acid-long protein containing 15 unique C-terminal amino acids, which are part of the L27 domain, without the PDZ, SH3 and GK domains (Figure 2b).

Phylogenetic analysis of the genomic sequence corresponding to the 3b(+) mRNAs suggests that these transcripts could be specifically expressed in primate tissues (Supplementary Table S2).

### Expression of human 3b(+) and 3b(−) *DLG1* mRNA in the post-mortem brains from patients with schizophrenia and bipolar disorder

**Figure 3:**
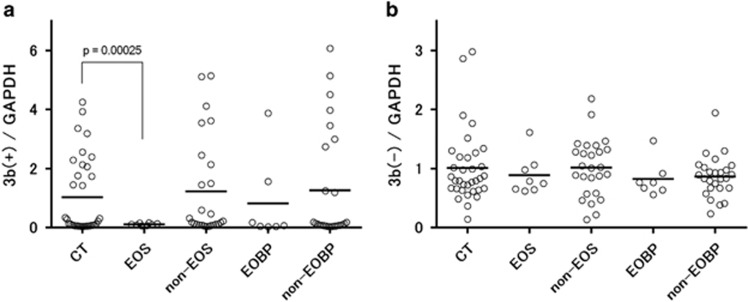
3b(+) and 3b(−) mRNA expression in the dorsolateral prefrontal cortices (DLPFCs) for each diagnostic group. (**a**) Expression of 3b(+) mRNA. A significant decrease in 3b(+) mRNA expression was observed in the EOS group compared with the control subjects (*P*=0.0003, Welch's *t*-test). There were no significant differences between each of the other patient groups and control group. CT, control (*n*=34); EOS, early-onset schizophrenia (*n*=8); non-EOS, non-early-onset schizophrenia (*n*=26); EOBPs, early-onset bipolar disorders (*n*=7); non-EOBPs, non-early-onset bipolar disorders (*n*=26). (**b**) Expression of 3b(−) mRNA. Expression levels were normalized to that of *GAPDH*. Horizontal bars indicate means.

Using qRT-PCR, we analyzed the 3b(+) and 3b(−) mRNA expression in Brodmann area 46 of the post-mortem DLPFC in each diagnostic group (Figure 3). Because we confirmed no significant differences in the levels of *GAPDH* mRNA in each diagnostic group compared with the controls in the study to examine the expression of housekeeping genes (Supplementary Table S1), the *GAPDH* mRNA values were used to normalize the data for 3b(+) and 3b(−). Although the 3b(−) mRNA expression had a normal distribution (Figure 3b), the distribution of the 3b(+) mRNA expression was positively skewed (Figure 3a).

No significant correlations were detected between the 3b(+) or 3b(−) mRNA expression levels and the age, post-mortem interval, brain pH or lifetime dose of antipsychotics. There was no significant difference in the 3b(+) or 3b(−) mRNA expression levels between the male and female subjects.

Only the EOS group (*n*=8) exhibited significantly reduced expression levels of 3b(+) mRNA compared to the controls (t=4.10, df=33.2, *P*=0.00025, Welch's t-test) (*P*<0.001 with the Bonferroni correction) (Figure 3a). Similar 3b(+) mRNA expression levels were observed in non-EOS (*n*=26) and EOBP (*n*=7) or non-EOBP (*n*=26) compared to the controls (*n*=34). In contrast, no significant difference in the 3b(-) mRNA expression was found between the controls and any of the other diagnostic groups (Figure 3b).

### Presence of exonic splicing enhancer consensus in exon 3b of human *DLG1* gene

**Figure 4:**
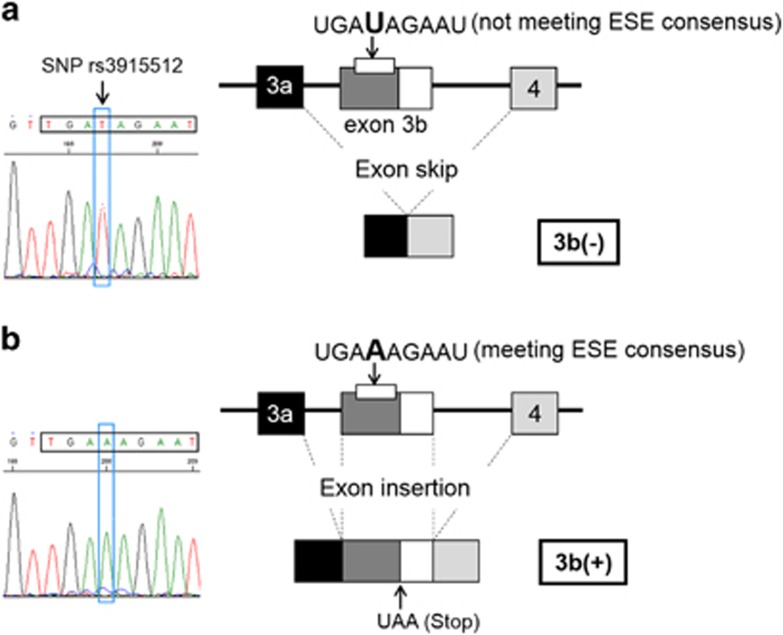
Outline of single-nucleotide polymorphism (SNP)-dependent insertion of exon 3b. (**a**) When the SNP rs3915512 consists of the T allele, the sequence (TGA**T**AGAAT) does not meet the exonic splicing enhancer (ESE) consensus. Therefore, exon 3b skipping would tend to occur, leading to the production of the known *DLG1* transcripts. (**b**) When the SNP consists of the A allele, the sequence (TGA**A**AGAAT) meets the ESE consensus, leading to the formation of an exon 3b-inserted splicing variant. The consensus of ESE was identified by Web analyses using ESEfinder (http://rulai.cshl.edu/tools/ESE/), FAS-ESS (http://genes.mit.edu/fas-ess/) and RESQUE-ESS (http://genes.mit.edu/burgelab/rescue-ese/).

An *in silico* structural analysis of exon 3b of the human *DLG1* gene using ESEfinder, FAS-ESS, and RESQUE-ESS indicated the presence of an exonic splicing enhancer (ESE) consensus (TGAAAGAAT) in exon 3b (Figure 4). This consensus sequence includes the SNP rs3915512 (underlined portion: TGA(A/T)AGAAT), the genotypes of which are found to be related to the expression levels of 3b(+) *DLG1* mRNA as described below.

### Relationships between genotype and post-mortem brain expression of human 3b(+) *DLG1* mRNA

**Figure 5:**
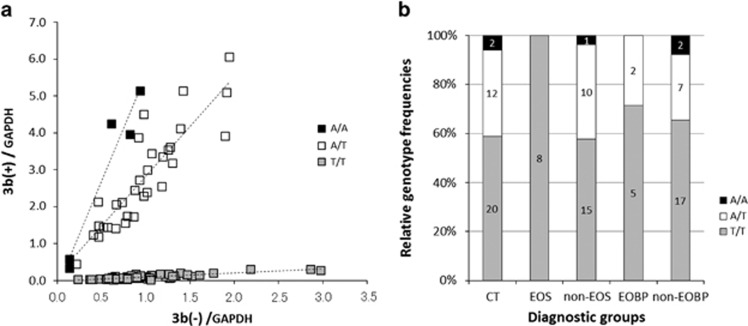
Expression of 3b(+) variant transcript depending on the genotype of single-nucleotide polymorphism (SNP) rs3915512. (**a**) All samples from each diagnostic group are plotted together in two dimensions. Whereas subjects with genotypes containing the A allele (A/A or A/T) demonstrated a certain amount of 3b(+) mRNA expression, subjects with the T/T genotype demonstrated an extremely low expression. There were positive correlations between the 3b(+) and the 3b(−) mRNA expression for each genotype (A/A: *n*=5, *r*=0.97, *P*<0.01, A/T: *n*=31, *r*=0.88, *P*<0.001, T/T: *n*=65, *r*=0.85, *P*<0.001). The 3b(−) mRNA expression appears to be independent of genotype. Expression levels were normalized to that of *GAPDH*. (**b**) Genotypic frequencies in each diagnostic group. Relative frequencies of each genotype are shown for each diagnostic group. The numerical values in the rectangular bars are the number of subjects with the corresponding genotypes. The early-onset schizophrenia group included no subjects with genotypes containing the A allele (A/A or A/T), whereas some (29–42%) of subjects in the other groups had genotypes containing the A allele.

The genotyping of the SNP rs3915512, which is the only SNP that has so far been reported in exon 3b, revealed that the genotypic and allelic frequencies of the CT group of the current study (A/A:A/T:T/T=0.06:0.35:0.59, A:T=0.24:0.76) are comparable to those of Utah Residents with Northern and Western European Ancestry in the dbSNP b126 data (A/A:A/T:T/T=0.06:0.50:0.44, A:T=0.31:0.69) of the International HapMap project (http://hapmap.ncbi.nlm.nih.gov/index.html.en). Individuals with the T/T genotype of SNP rs3915512 demonstrated extremely low levels of cortical 3b(+) mRNA expression, whereas those with the A/A and A/T genotypes showed moderate to high levels (Figure 5a). Moreover, no subjects in the EOS group possessed genotypes containing the A allele (A/A or A/T) in the above SNP, whereas some (29–42%) of the subjects in the other four groups had the A allele in their genotypes (Figure 5b).

There were positive correlations between the 3b(+) and 3b(−) mRNA expression for each genotype (Figure 5a). However, when considering only the subjects with the T/T genotype, there was no correlation between the 3b(+) and 3b(−) mRNA expressions in the EOS group, whereas other diagnostic groups demonstrated positive correlations (Supplementary Figure S2).

The expression of 3b(−) mRNA was found to be independent of genotype.

### Secondary analysis: association between suicide status and human 3b(+) expression or genotype

As a secondary analysis, we investigated whether the suicide status is associated with the 3b(+) expression or the genotype of SNP rs3915512. The number of suicides is shown in Supplementary Table S1. When including controls, there was no significant difference in the 3b(+) levels between subjects who committed suicide (suicide completers) and those who died from reasons other than suicide (non-suicide; 1.39 compared with 0.96, *t*=−0.94, df=25.8, *P*=0.36, Welch's *t*-test). There was no significant difference in genotype frequencies between the suicide completers (A/A: 2, A/T: 7, T/T: 12) and non-suicides (A/A: 3, A/T: 24, T/T: 53; *P*=0.44, Fisher's exact test). Similar results were also obtained when the controls were not included; there was no significant difference in the 3b(+) levels between the suicide completers and non-suicides (1.39 compared with 0.91, *t*=−0.98, df=31.5, *P*=0.33, Welch's *t*-test) or in the genotype frequencies between the suicide completers (A/A: 2, A/T: 7, T/T: 12) and non-suicides (A/A: 1, A/T: 12, T/T: 33*; P*=0.28, Fisher's exact test).

### Deposition of accession numbers

The 3b(+) variants were designated *Homo sapiens* discs, large homolog 1 (Drosophila) (*DLG1*), transcript variants 6-v1 (A allele or Lys allele) and 6-v2 (T allele or Ile allele) and were deposited as AB855790 and AB855791, respectively, in the DNA Data Bank of Japan (DDBJ, http://www.ddbj.nig.ac.jp/index-e.html).

## Discussion

In the present study, we have newly isolated a splicing variant, 3b(+) mRNA, of the human *DLG1* gene that is mapped to chromosome 3q29, a schizophrenia-susceptibility locus, in humans. Post-mortem examination revealed that the mRNA expression level of the 3b(+) variant is lower in the DLPFC of the EOS group but not in the non-EOS, EOBP or non-EOBP groups compared with the CT group and that the 3b(+) *DLG1* mRNAs are expressed in the brain tissue in a SNP rs3915512 genotype-dependent manner. Furthermore, there is a positive correlation between the 3b(−) and 3b(+) mRNA expression for the subjects with the T/T genotype of SNP rs3915512 in every diagnostic group except the EOS group. Together with the presence of the ESE consensus in exon 3b that contains a SNP affecting the splicing function, these results indicated that the newly identified 3b(+) mRNA of the *DLG1* gene, which encodes a glutamate transmission-related molecule, could be under specific regulation and be dysregulated in the brains of the EOS group.

The reduced expression of the 3b(+) mRNA in the cortex of one diagnostic group could be because of a nonspecific phenomenon related to certain problems of sample quality. However, this possibility is argued against by following facts: (i) the homogeneity with regard to the RNA quality, (ii) no correlation between the changes in the 3b(+) mRNA expression levels and the values of each index affecting the RNA quality in the currently used post-mortem brain samples and (iii) no significant alterations in the prefrontal 3b(−) mRNA expression for the EOS group (argued in 'Additional explanations and discussions for Supplementary Table S1').

A sequence analysis of the 3b(+) mRNA suggests that because of an in-frame stop codon in the mRNA, the translation of this transcript would result in a truncated DLG1 protein without a third alpha-helix in the N-terminal L27 domain or the PDZ, SH3 and GK domains (Figure 2). The putative truncated protein would modify the protein assemblies of various known DLG1 proteins (Figure 2). Because of the lack of known complete domains, it cannot be excluded that the 3b(+) transcript could function as a long-noncoding RNA^30^ that might control the transcription of the L27 domain sequence within the *DLG1* mRNA. Alternatively, the presence of an in-frame stop codon in the exon 3b insertion suggests the possible regulation of the 3b(+) transcripts by a nonsense-mediated mRNA decay pathway^31^ to eliminate them as an error expression or to preserve the normal homeostasis of transcription of the 3b(+) variants. The latter maintenance role has recently been raised by modification experiments of nonsense-mediated mRNA decay factors in cell lines and mice and been considered to be related to human diseases.^31^ This role for the 3b(+) transcript could be reflected in the fact that there are large interindividual variations in the expression levels of the cortical 3b(+) transcript, that is, the ratio of their maximum to minimum values in the present assay is greater than two hundred (Figure 3a). In addition, the higher values of the 3b(+) expression are at least near the range of the protein-coding mRNA expression in terms of the PCR amplification cycles (Figures 1b and 3a).

Whatever the mechanisms through which the 3b(+) transcript functions in the brain, the results of this study indicated the fine controls on the 3b(+) variant expression that might have an important role in the interactions among the various *DLG1* transcripts. Thus, the expression levels of the cortical 3b(+) mRNA are closely correlated to the genotype of the SNP rs3915512 located in the sequence of the ESE (TGA(A/T)AGAAT) (Figure 4). The genotype-dependent expression of the 3b(+) mRNA is likely to be caused by an exon insertion/skipping mechanism. When the T allele of the SNP is expressed, the sequence (TGA**T**AGAAT) does not meet the ESE consensus. Therefore, exon 3b skipping would tend to occur, leading to the preference for the production of the known 3b(−) *DLG1* transcripts (Figure 4a). Thus, the subjects in the EOS group, all of whom have the T/T genotype, exhibited extremely low levels of 3b(+) mRNA expression. In contrast, the sequence containing the A allele of the SNP meets the ESE consensus and would result in the formation of an exon 3b insertion splicing variant (Figure 4b). Furthermore, there is a positive correlation between the 3b(−) and the 3b(+) mRNA expression for the T/T genotype group of the SNP rs3915512 in every diagnostic group except the EOS group. This positive correlation indicated the coordinated expression of the *DLG1* gene transcripts and also the existence of certain regulatory factors for this coordination besides the above mentioned SNP in the ESE because an inverse correlation between the 3b(−) and the 3b(+) mRNA expression would be expected if the SNPs were the only determinant for the alternative expression of the 3b(−) or 3b(+) mRNA. Consequently, the lack of a significant correlation between the 3b(−) and the 3b(+) mRNA expression in the present EOS patients could be because of deviance from the physiological coordination of the *DLG1* variant expression.

Because exon 3b is transcribed into a part of the *DLG1* mRNA that would otherwise be translated as a portion of the L27 domain (Figure 2) and because the L27 domain acts as an organization center for the formation of a DLG1 oligomer,^12, 13^ the 3b(+) mRNA could participate in control of the glutamate neurotransmission through modulation of the assemblage of various 3b(−) mRNA-associated DLG1 proteins that interact with ionotropic glutamate receptors through their PDZ domains. Altered expression of the 3b(+) mRNA could therefore cause changes in the expression of the DLG1 proteins and/or of the *DLG1* mRNAs that have been found in previous post-mortem studies,^16, 17^ although there are some inconsistencies among these studies, which might be because the precise brain areas in the DLPFC are different or because of the heterogeneity introduced by factors including age of onset that are different among the brain banks.

In this regard, the putative truncated protein might affect the trafficking of AMPAR and its stabilization in the synaptic membrane, which is an essential role of DLG1. A study investigating the protein structure and function of DLG1 revealed that the synaptic localization of the AMPAR requires L27-mediated multimerization of DLG1.^32^ It is inferred that the truncated protein, at least partially, retains the function of the L27 domain and might alter the equilibrium of DLG1 in AMPAR trafficking. Several studies have reported changes in the expression of molecules associated with AMPAR trafficking in schizophrenia.^33, 34^ In our previous studies, as we described in the introduction, we demonstrated that the mRNA expression of *DLG1* is increased in response to phencyclidine only after a critical stage of development and hypothesized that a human brain system equipped with a certain molecular cascade is responsible for this phenomenon. We would propose that AMPA trafficking is involved in such a molecular cascade mediated by NMDAR and that the functional maturation of the cascade is required for *DLG1* to respond to phencyclidine in a development-dependent manner. The putative truncated protein *DLG1* 3b(+) might have a ‘dominant interfering' action on AMPAR trafficking, and those with a lower level of *DLG1* 3b(+), such as subjects in the EOS group, may have a faster maturation of that system, thus developing the first symptoms earlier than those who have adequate levels of *DLG1* 3b(+). However, it appears that the lack of 3b(+) is not the only factor that contributes to the earlier onset because a substantial portion of subjects in the non-EOS, EOBP, non-EOBP and CT groups has a lower level of 3b(+). For those with a lower level of 3b(+), but non-early onset, there might be compensatory mechanisms that regulate the AMPA trafficking. The similar level of the 3b(−) mRNA expression across the diagnostic groups in the current study is consistent with a study conducted by another research group.^35^

In conclusion, our findings have demonstrated that there is an EOS-specific reduction in the expression of the unreported 3b(+) *DLG1* mRNA in Brodmann area 46 of the DLPFC and that the expression levels of the 3b(+) *DLG1* mRNA depend on the genotype of SNP rs3915512 located in the ESE site in the newly identified exon 3b that is required for transcription of the 3b(+) mRNA. Together with the significant role of the *DLG1* gene and its protein products in glutamatergic transmission, these data are consistent with the view that the decreased 3b(+) expression and the genotype of the SNP rs3915512 might be involved in the pathophysiology and/or susceptibility of a group of EOS. However, the current study is limited in that the functional role of 3b(+) transcript/protein or the influence of genotype of SNP rs3915512 on NMDA/AMPA receptors has not been established. A functional analysis of the 3b(+) transcript/protein and an onset age-stratified genetic association analysis between the SNP and schizophrenia are currently in progress.

## Notes

These authors declare no conflict of interest.
