# Supplementary material for: Reduced cortical expression of a newly identified splicing variant of the DLG1 gene in patients with early-onset schizophrenia
Source: Transl Psychiatry. 2015 Oct 6;5(10):e654–. doi: 10.1038/tp.2015.154 (PMC4930131; doi:10.1038/tp.2015.154)
Supplement: Supplementary Information [file tp2015154x1.pdf]

Supplementary Information

Supplementary Table S1. Demographic characteristics of the control and each diagnostic group

Variables	CT	EOS	non-EOS	EOBP	non-EOBP	*P* value	Post hoc primary findings (*P* value)[d]
**Number**	**n = 34**	**n = 8**	**n = 26**	**n = 7**	**n = 26**		
**Age (yrs)[a]**	44.1 ± 7.68	44.6 ± 8.26	42.1 ± 8.71	36.9 ± 11.9	47.8 ± 9.45	0.034[b]	EOBP < non-EOBP (0.03)
**Onset Age[a]**	-	14.4 ± 2.61	23.6 ± 5.17	15.3 ± 1.38	28.2 ± 8.56	$4.14x10^{-7}$[b]	EOS < non-EOS (0.004), EOBP < non-EOBP (< 0.001)
**Male/Female**	25/9	5/3	20/6	4/3	11/15	0.62[c]	
**PMI (hours)[a]**	29.3 ± 13.1	41.3 ± 17.0	28.2 ± 14.3	29.6 ± 15.1	39.2 ± 18.4	0.03[b]	non-EOS < non-EOBP (0.08)
**Brain pH[a]**	6.61 ± 0.27	6.66 ± 0.16	6.41 ± 0.24	6.4 ± 0.29	6.42 ± 0.31	0.009[b]	CT > non-EOS (0.04)
**Housekeeping gene expression[e]**							
GAPDH	28.6 ± 16.6	28.3 ± 18.7	22.8 ± 12.7	23.5 ± 22.7	19.2 ± 12.7	0.18[b]	
ACTB	52.3 ± 28.9	52.2 ± 29.3	43.8 ± 25.3	45.2 ± 38.9	35.9 ± 23.8	0.18[b]	
PGK1	29 ± 14.7	28.1 ± 17.4	22.3 ± 13.0	23.1 ± 20.8	20.1 ± 13.8	0.17[b]	
**rRNA Content (%)[f]**							
**18S rRNA**	7.50 ± 1.51	7.76 ± 1.35	7.37 ± 1.49	7.02 ± 1.83	7.36 ± 1.89	0.93[b]	
**28S rRNA**	9.10 ± 3.93	8.31 ± 2.26	8.82 ± 3.55	7.00 ± 4.40	8.12 ± 4.85	0.73[b]	
**Lifetime Fluphenazine equivalent (mg)[a]**	-	105,006 ± 136,481	815,42 ± 90,218	7,857 ± 10,156	11,448 ± 26,137	0.001[b]	non-EOBP < EOS (0.02), non-EOS (0.008)
**Number of suicides**	0	0	7	2	12		

There were significant differences among the diagnostic groups in age (EOBP < non-EOBP), onset age (EOS < non-EOS, EOBP < non-EOBP, as expected), postmortem interval (PMI) (non-EOS < non-EOBP), brain pH (CT > non-EOS), and lifetime dose of antipsychotics (non-EOBP < EOS, non-EOS, excluding controls). There was no significant difference in the gender distributions. Abbreviations: CT, controls; EOS, early-onset schizophrenia; non-EOS, non-early-onset schizophrenia; EOBP, early-onset bipolar disorder; non-EOBP, non-early-onset bipolar disorder; PMI, postmortem interval. [a]mean ± standard deviation, [b]One-way Analysis of Variance, [c]Fisher's exact test, [d]Tukey's multiple comparison test, [e]Arbitrary unit, [f]Denominator is total RNA

Additional explanations and discussions for Supplementary Table S1

In this supplementary section, further evaluations of the quality of RNAs in the postmortem brain tissues used in the present study have been completed.

Brain pH has been a widely accepted index of brain tissue quality. Recently, the RIN value has been recognized to be a better marker for RNA integrity of postmortem brain tissues. Nonetheless, there are still no absolute indices to justify the quality. In fact, the brain samples of several diagnostic groups have been reported to show significantly lower pH values than other groups without indicating significantly lower RIN values in a large brain collection.[1] The 5'/3' ratio of housekeeping genes and histological assessment of the cerebellar granule cell layer have been proposed as candidate indices for RNA and tissue integrity, respectively.[2, 3]

We obtained total RNA from the postmortem brain tissues provided by the SMRI and measured several markers of their quality including the pH, 18S and 28S rRNA, expression of a housekeeping gene, *GAPDH*, but not RIN values, because the RIN value was not widely recommended as a better marker of postmortem brain samples at that time. The pH values of our brain samples (Supplementary Table S1) were within the acceptable range for reliable RT-PCR assay of mRNA expression that is seen as an example in a very recent paper.[4] Indeed, our data from the RT-PCR measures using the same samples from the SMRI have been accepted by the following authoritative scientific journals: Biological Psychiatry, Molecular Psychiatry, Schizophrenia Research, Journal of Neurochemistry, Proceedings of the National Academy of Sciences, American Journal of Medical Genetics Part B, Human Genetics, and Progress in Neuro-Psychopharmacology and Biological Psychiatry, in that the quality of the brain samples as well as the assay methodologies have been recognized as being reliable.[5-12]

Importantly, it is generally noted that sample sets should be matched with regard to RNA quality and that no correlation between the changes in the target mRNA expression levels and the values of an RNA quality index should be detected. As shown in Supplementary Table S1, an ANOVA analysis followed by a post hoc test indicates significant differences in the pH values only between the control and non-early-onset schizophrenia (non-EOS) groups. However, similar expression levels of the housekeeping genes, *GAPDH, ACTB, PGK1*, 18S and 28S rRNA, observed across all the diagnostic groups suggest that the samples maintain the homogeneity of their RNA quality in the present sample set. Moreover, the fact that no significant correlation between the pH values and the expression levels of 3b(+) or 3b(-) transcript was detected in the diagnostic group is consistent with the idea that the measurement of 3b(+) and 3b(-) transcription is not influenced by the brain pH. Finally, the 3b(+) data are associated with genotype, whereas there were no significant correlations between the 3b(+) transcript levels and age, brain pH, or PMI for any of the brain samples and no difference in the 3b(-) mRNA expression among the groups was found. These data thus allow us to consider that the selectively altered expression of the 3b(+) transcript in the EOS group does not depend on these indices affecting brain sample quality.

The papers necessary for the aforesaid disputation have been presented below.

1. Webster MJ. Tissue preparation and banking. *Prog Brain Res* 2006; **158**: 3-14.

2. Popova T, Mennerich D, Weith A, Quast K. Effect of RNA quality on transcript intensity levels in microarray analysis of human post-mortem brain tissues. *BMC genomics* 2008; **9**: 91.

3. Sheedy D, Harding A, Say M, Stevens J, Kril JJ. Histological assessment of cerebellar granule cell layer in postmortem brain; a useful marker of tissue integrity? *Cell and tissue banking* 2012; **13**(4): 521-527.

4. Chandley MJ, Szebeni A, Szebeni K, Crawford JD, Stockmeier CA, Turecki G*, et al.* Elevated gene expression of glutamate receptors in noradrenergic neurons from the locus coeruleus in major depression. *Int J Neuropsychopharmacol* 2014; **17**(10): 1569-1578.

5. Aoki-Suzuki M, Yamada K, Meerabux J, Iwayama-Shigeno Y, Ohba H, Iwamoto K*, et al.* A family-based association study and gene expression analyses of netrin-G1 and -G2 genes in schizophrenia. *Biol Psychiatry* 2005; **57**(4): 382-393.

6. Kato T, Iwayama Y, Kakiuchi C, Iwamoto K, Yamada K, Minabe Y*, et al.* Gene expression and association analyses of LIM (PDLIM5) in bipolar disorder and schizophrenia. *Mol Psychiatry* 2005; **10**(11): 1045-1055.

7. Shimizu H, Iwayama Y, Yamada K, Toyota T, Minabe Y, Nakamura K*, et al.* Genetic and expression analyses of the STOP (MAP6) gene in schizophrenia. *Schizophr Res* 2006; **84**(2-3): 244-252.

8. Ide M, Ohnishi T, Murayama M, Matsumoto I, Yamada K, Iwayama Y*, et al.* Failure to support a genetic contribution of AKT1 polymorphisms and altered AKT signaling in schizophrenia. *J Neurochem* 2006; **99**(1): 277-287.

9. Yamada K, Gerber DJ, Iwayama Y, Ohnishi T, Ohba H, Toyota T*, et al.* Genetic analysis of the calcineurin pathway identifies members of the EGR gene family, specifically EGR3, as potential susceptibility candidates in schizophrenia. *Proc Natl Acad Sci U S A* 2007; **104**(8): 2815-2820.

10. Anitha A, Nakamura K, Yamada K, Iwayama Y, Toyota T, Takei N*, et al.* Association studies and gene expression analyses of the DISC1-interacting molecules, pericentrin 2 (PCNT2) and DISC1-binding zinc finger protein (DBZ), with schizophrenia and with bipolar disorder. *Am J Med Genet B Neuropsychiatr Genet* 2009; **150B**(7): 967-976.

11. Yamada K, Iwayama Y, Toyota T, Ohnishi T, Ohba H, Maekawa M*, et al.* Association study of the KCNJ3 gene as a susceptibility candidate for schizophrenia in the Chinese population. *Human genetics* 2012; **131**(3): 443-451.

12. Bangel FN, Yamada K, Arai M, Iwayama Y, Balan S, Toyota T*, et al.* Genetic analysis of the glyoxalase system in schizophrenia. *Prog Neuropsychopharmacol Biol Psychiatry* 2015; **59**: 105-110.

Supplementary Table S2. Phylogenic sequence homology across vertebrates of a *DLG1* 3b(+) variant

Species	General name	BLAST Max score	Accession	Nucleotide homology in exon 3b with human
Homo sapiens	Human	242	NC_018914.2	95/95
Homo sapiens	Human	242	NC_000003.12	94/95 (isoform x4, SNP)
Pongo abelii	Sumatran orangutan	237	NC_012594.1	89/95
Pan troglodytes	Chimpanzee	237	NC_006490.3	94/95
Pan paniscus	Bonobo	237	NW_003870089.1	91/95
Gorilla gorilla	Gorilla	237	NC_018427.1	91/95
Macaca mulatta	Rhesus macaque	237	NC_007859.1	89/95
Macaca fascicularis	Crab-eating macaque	237	NC_022273.1	89/95
Ceratotherium simum	White rhinoceros	233	NW_004454169.1	76/95
Mustela putorius	European polecat	233	NM_004577124.1	69/95
Callithrix jacchus	Common marmoset	233	NC_013910.1	85/95
Nomascus leucogenys	White-cheeked gibbon	233	NC_19826.1	90/95
Papio anubis	Olive baboon	230	NC_018153,1	78/95
Equus caballus	Horse	228	NC_009162.2	72/95
Felis catus	Domestic cat	228	NC_018731.1	68/95
Bos taurus	Cattle	219	NC_007299.5	No similarity
Mus musculus, C57BL/6J	Mouse	210	NC_000082.6	No similarity
Rattus norvegicus	Rat	201	NC_005110.4	No similarity

BLASTN search was performed for 3b(+) cDNA with the NCBI genome database. Highly conserved genetic sequence similarity with the human exon 3b is found in nonhuman primates but not in rodents. The Max score indicates an alignment score of the overall transcript (Accession number AB855790) based on the BLAST search.

Supplementary Figure S1. Linear correlations among housekeeping genes

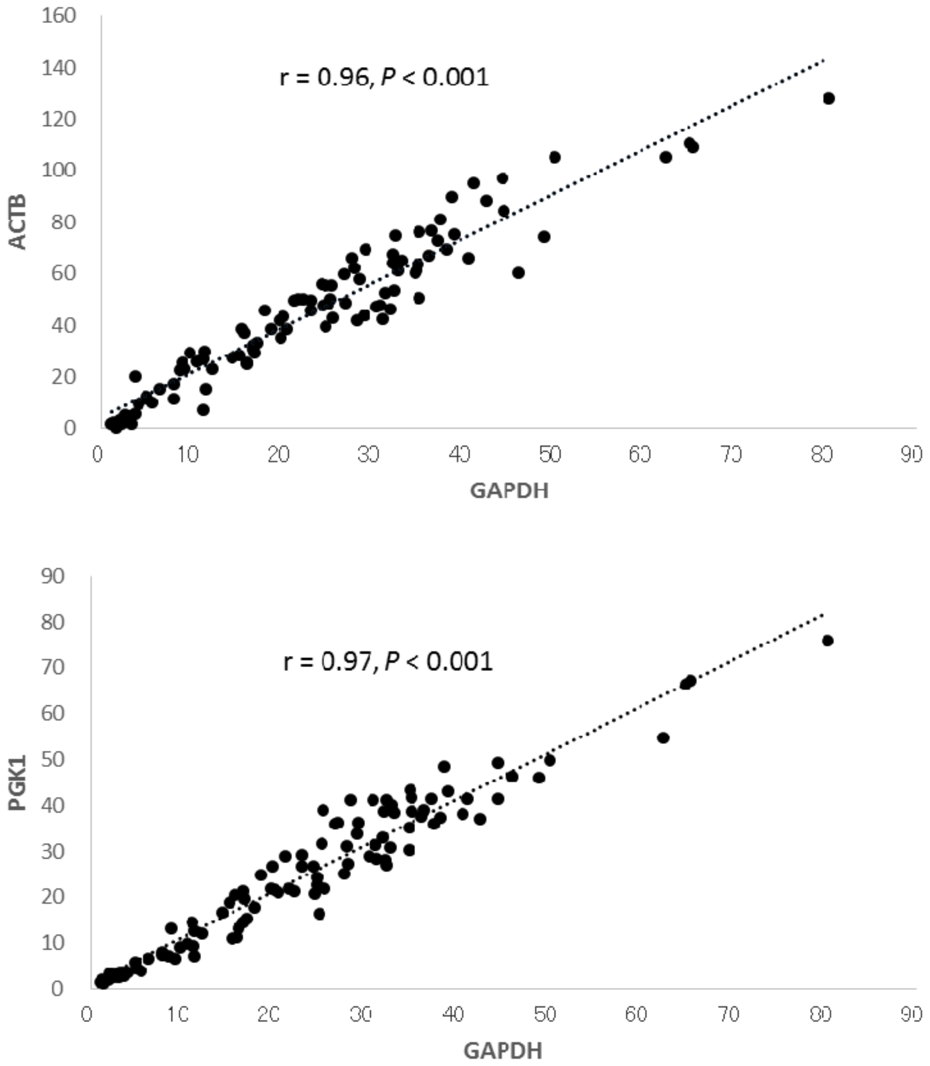

Using the same sample set, the expression of the housekeeping genes, *GAPDH, ACTB, and PGK1*, were measured to examine their correlations. The expression level of *GAPDH* is linearly correlated with those of other housekeeping genes, *ACTB* and *PGK1*.

Supplementary Figure S2. Correlations between 3b(+) and 3b(-) mRNA in subjects with the T/T genotype in each diagnostic group

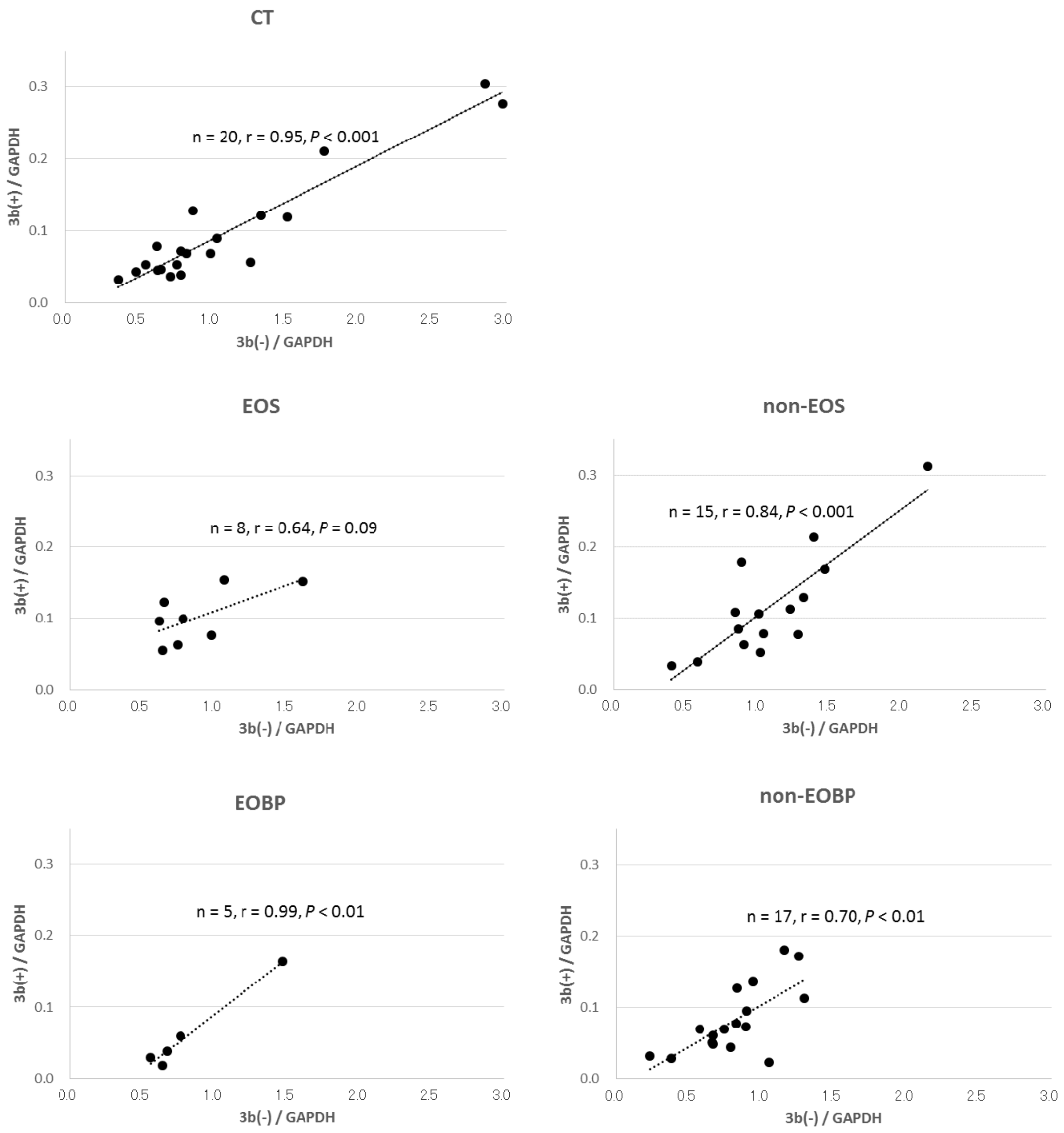

For subjects with the T/T genotype, there was no correlation between the expression of 3b(+) and 3b(-) mRNA in the EOS group, although other diagnostic groups demonstrated positive correlations (controls: n = 20, r = 0.95, $P < 0.001$, EOS: n = 8, r = 0.64, $P = 0.09$, non-EOS: n = 15, r = 0.84, $P < 0.001$, EOBP: n = 5, r = 0.99, $P < 0.01$, non-EOBP: n = 17, r = 0.70, $P < 0.01$).
